# Supplementary material for: Stable pure-green organic light-emitting diodes toward Rec.2020 standard
Source: Nat Commun. 2024 May 23;15:4394. doi: 10.1038/s41467-024-48659-6 (PMC11116534; doi:10.1038/s41467-024-48659-6)
Supplement: Supplementary file 1 — Supplementary Information [file 41467_2024_48659_MOESM1_ESM.pdf]

Stable pure-green organic light-emitting diodes toward Rec.2020 standard

Xun Tang[1]*, Tuul Tsagaantsooj[1], Tharindu P. B. Rajakaruna[1], Kai Wang[2], Xian-Kai Chen[2], Xiao-Hong Zhang[2], Takuji Hatakeyama[3], and Chihaya Adachi[1,4]*

[1]Center for Organic Photonics and Electronics Research (OPERA), Kyushu University, 744 Motooka, Nishi-ku, Fukuoka 819-0395, Japan.
[2]Institute of Functional Nano & Soft Materials (FUNSOM), Joint International Research Laboratory of Carbon-Based Functional Materials and Devices, Soochow University, Suzhou, Jiangsu 215123, P.R. China.
[3]Department of Chemistry, Graduate School of Science, Kyoto University, Kitashirakawa Oiwake-cho, Sakyo-ku, Kyoto, 606-8502 Japan.
[4]International Institute for Carbon-Neutral Energy Research (I2CNER), Kyushu University, 744 Motooka, Nishi-ku, Fukuoka 819-0395, Japan.

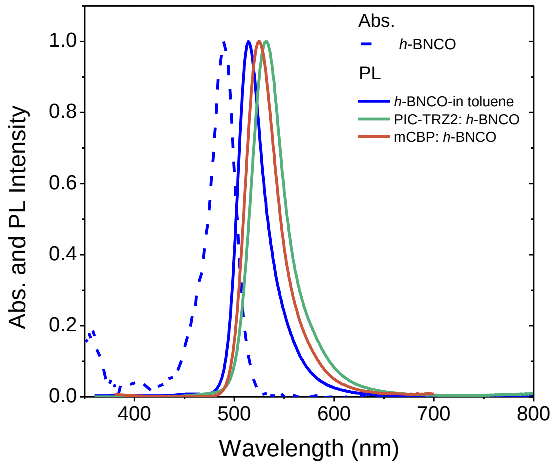

Supplementary Figure 1: Absorption and photoluminescence (PL) of *h*-BNCO in toluene. PL spectra of 1 wt% *h*-BNCO in PIC-TRZ2 and mCBP blend films.

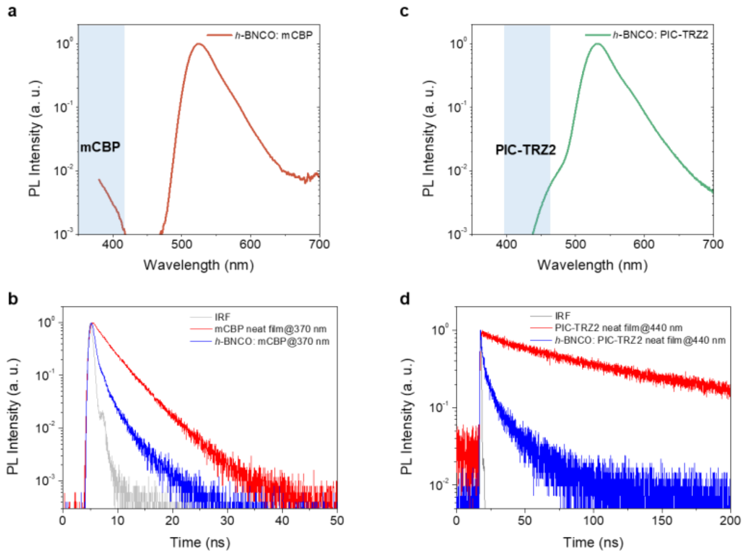

Supplementary Figure 2: PL spectrum of **a** 1 wt% *h*-BNCO: mCBP and **c** 1 wt% 1 wt% *h*-BNCO: PIC-TRZ2 films. Transient PL decay curve of **b** mCBP neat film and 1 wt% *h*-BNCO: mCBP blend film at 370 nm; **d** PIC-TRZ2 neat film and 1 wt% *h*-BNCO: PIC-TRZ2 blend film at 440 nm.

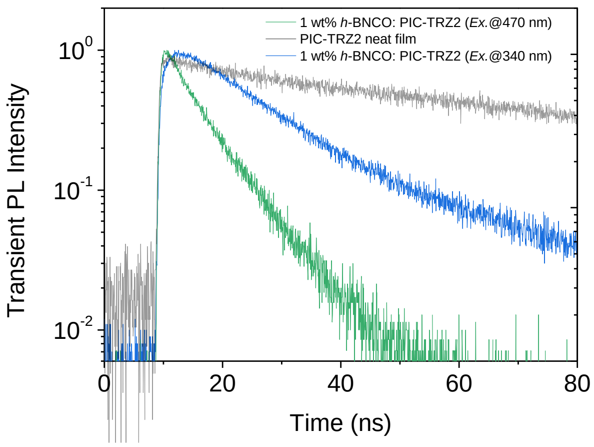

Supplementary Figure 3: Transient PL decay properties of a 1 wt% *h*-BNCO: PIC-TRZ2 film under the excitation wavelengths of 340 and 470 nm, respectively. Transient PL decay properties of a PIC-TRZ2 neat film under the excitation wavelengths of 340 nm.

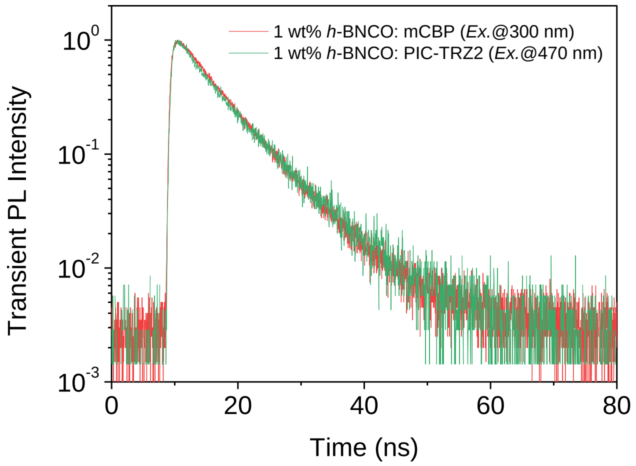

Supplementary Figure 4: Transient PL decay properties of a 1 wt% *h*-BNCO: mCBP film and a 1 wt% *h*-BNCO: PIC-TRZ2 film with the excitation wavelength of 300 nm and 470 nm, respectively.

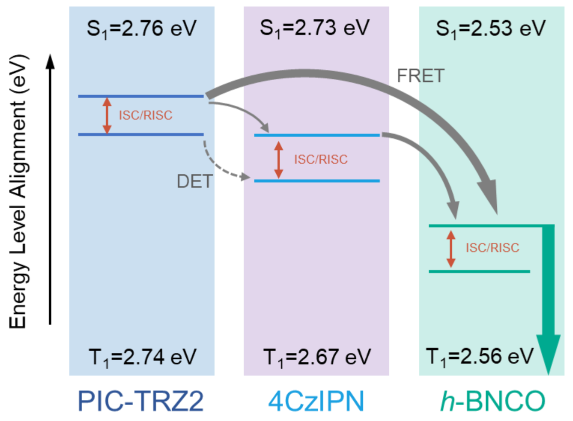

Supplementary Figure 5: Energy level alignment and energy transfer process in 1 wt% *h*-BNCO: 8 wt% 4CzIPN: PIC-TRZ2 blend film.

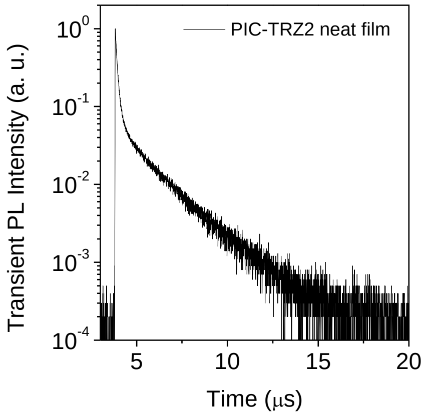

Supplementary Figure 6: Transient PL decay profile of a PIC-TRZ2 neat film.

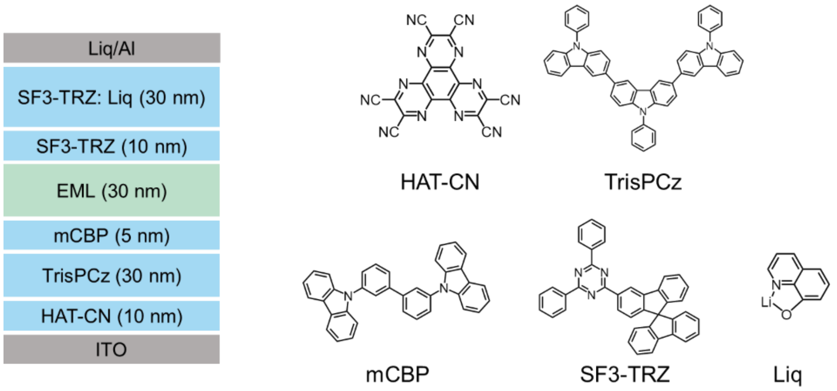

Supplementary Figure 7: The OLED structure and the molecular structures of functional layers.

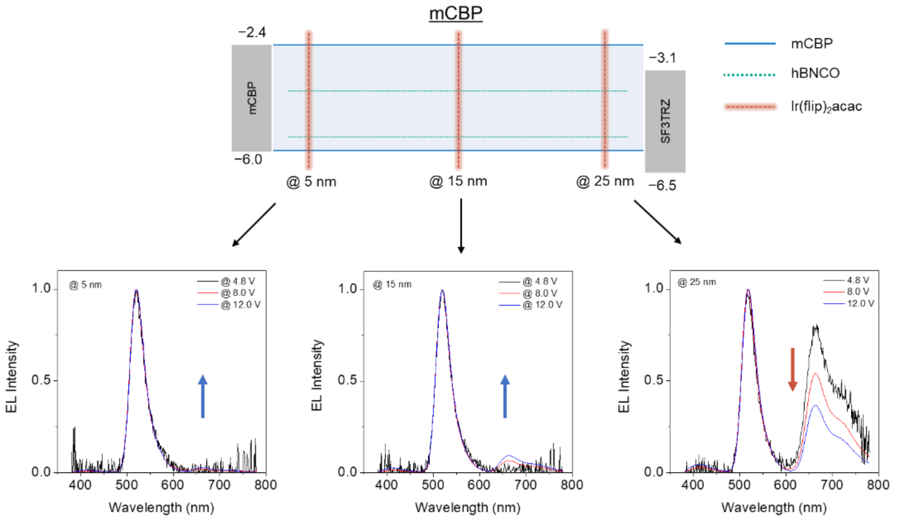

Supplementary Figure 8: The EL intensity of D1 with ultra-thin Ir(flip)2acac layers at different positions in the emissive layer (EML).

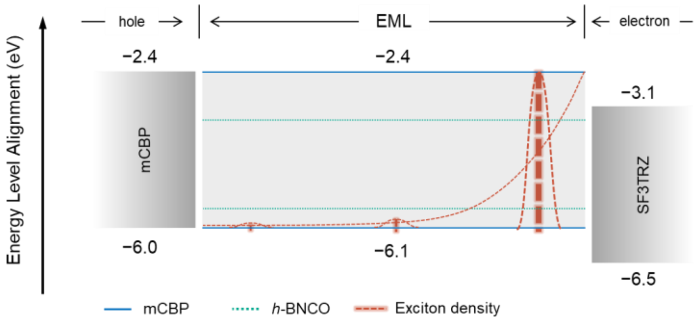

Supplementary Figure 9: The distribution of the recombination zone in D1.

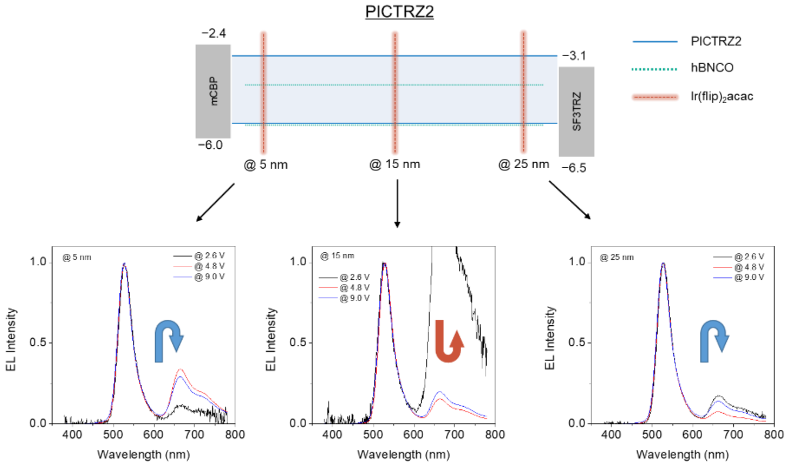

Supplementary Figure 10: The EL intensity of D2 with ultra-thin Ir(flip)2acac layers at different positions in the emissive layer (EML).

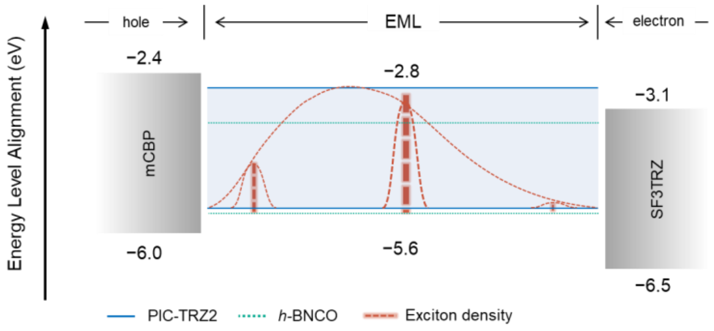

Supplementary Figure 11: The distribution of the recombination zone in D2.

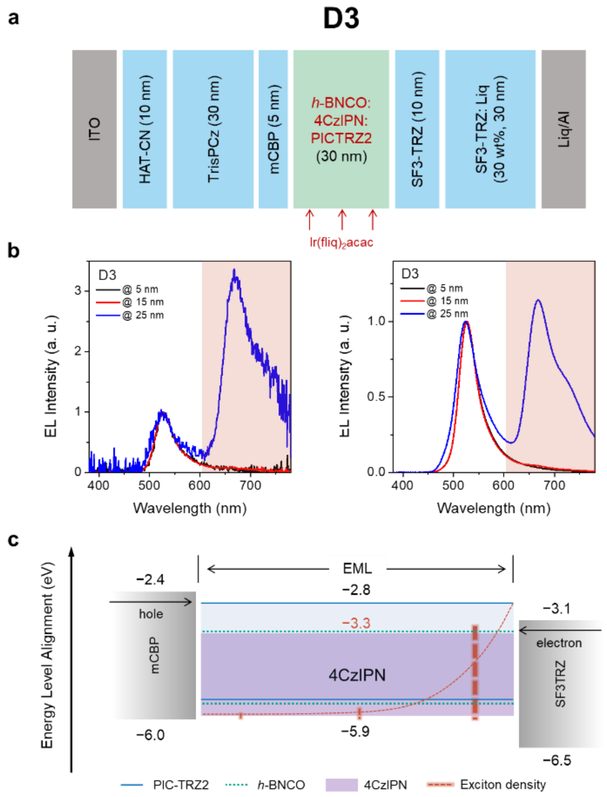

Supplementary Figure 12: a Device structure of D3 with Ir(fliq)$_2$acac films at 5, 15, and 25 nm of the emitting layer (EML). **b** EL spectra of D3 with Ir(fliq)$_2$acac at different positions in EML under low/high current density. **c** Illustration of the exact recombination zone distribution in D3.

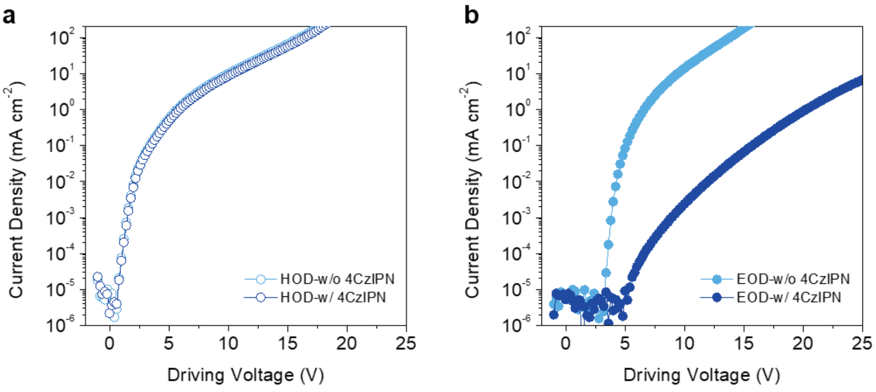

Supplementary Figure 13: a HOD and **b** EOD of D2 (1 wt% *h*-BNCO: PIC-TRZ2) and D3 (1 wt% *h*-BNCO: 8 wt% 4CzIPN: PIC-TRZ2).

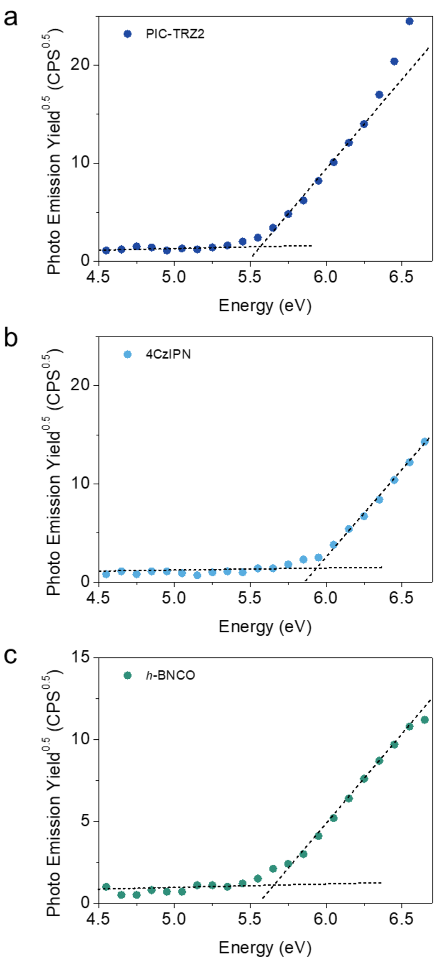

Supplementary Figure 14: Photoelectron yield spectra of **a** PIC-TRZ2, **b** 4CzIPN, and **c** *h*-BNCO, which were measured by a spectrometer (AC-3, Riken Keiki).

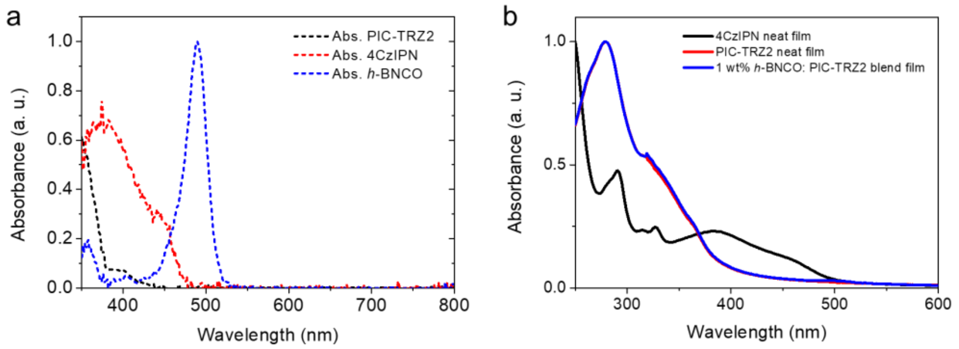

Supplementary Figure 15: a Absorption bands of PIC-TRZ2, 4CzIPN, and *h*-BNCO in toluene. **b** Absorption bands of PIC-TRZ2 and 4CzIPN neat films, and the 1 wt% h-BNCO:PIC-TRZ2 blend film.

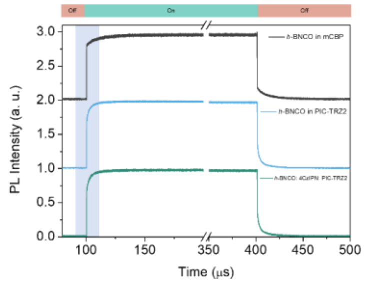

Supplementary Figure 16: Transient PL emission characteristics under pulsed excitation of 1 wt% *h*-BNCO: mCBP, 1 wt% *h*-BNCO: PIC-TRZ2, and 1 wt% *h*-BNCO: 8 wt% 4CzIPN: PIC-TRZ2 blend films.

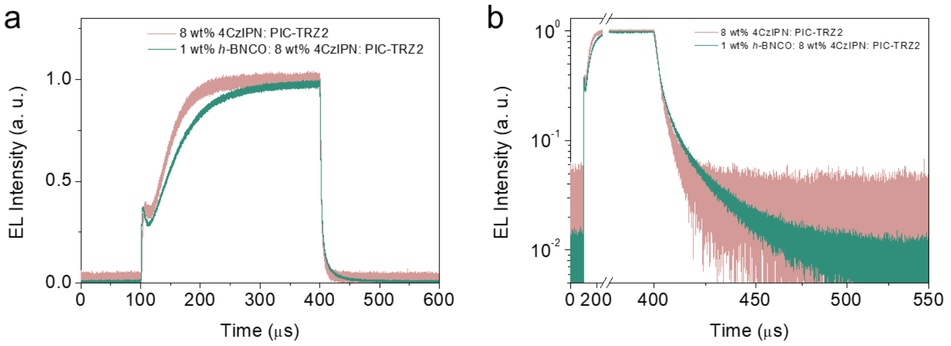

Supplementary Fig. 17: a Transient EL decay properties of D3 and D5, **b** transient EL delayed emission properties under a pulse width of 300 μs. The EML of D5 is 8 wt% 4CzIPN: PIC-TRZ2.

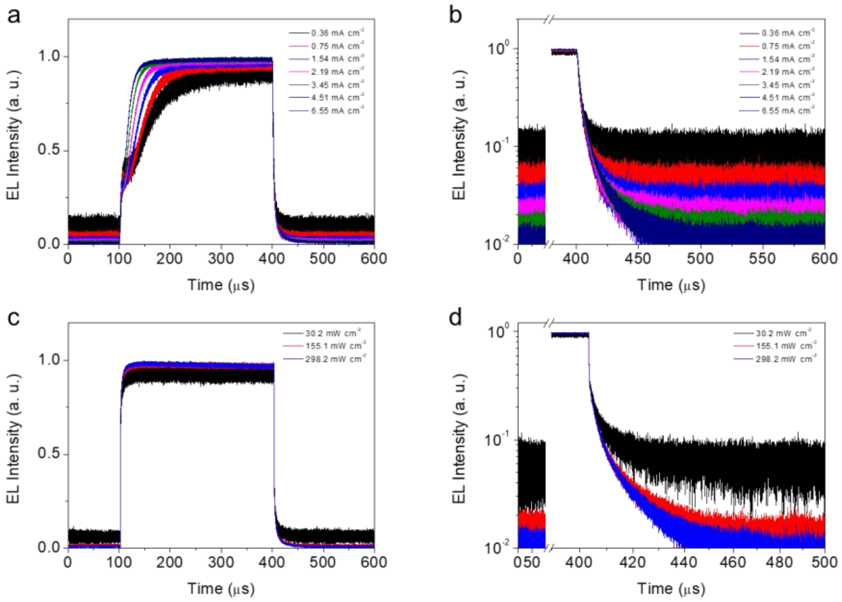

Supplementary Figure 18: a Transient EL decay properties of D5, and **b** transient EL delayed emission properties under a pulse width of 300 μs and different current densities. **c** Transient PL decay properties of D5, and **d** transient PL delayed emission properties under a pulse width of 300 μs and different excitation powers.

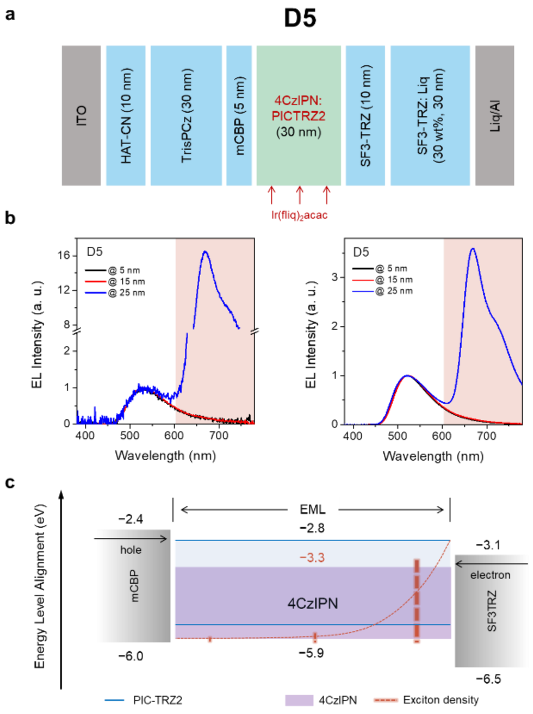

Supplementary Figure 19: a Device structure of D5 with Ir(fliq)2acac films at 5, 15, and 25 nm of the emitting layer (EML). **b** EL spectra of D5 with Ir(fliq)2acac at different positions in EML under low/high current density. **c** Illustration of the exact recombination zone distribution in D5.

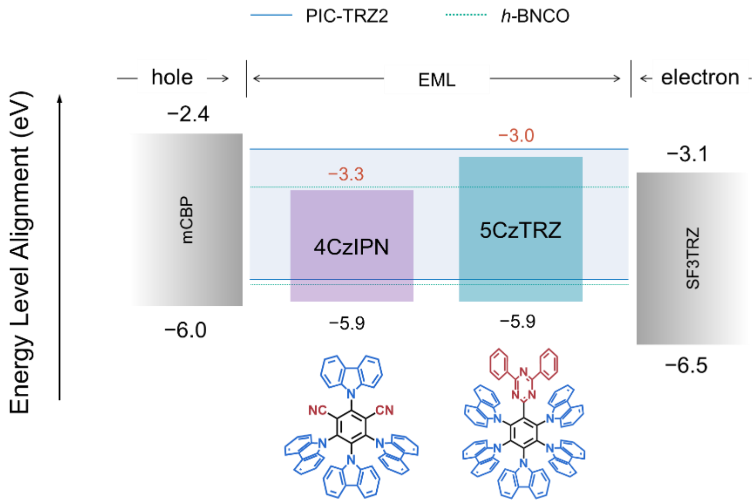

Supplementary Figure 20: Energy level alignments of 4CzIPN-based OLED (D3) and 5Cz-TRZ-based OLED (D6). The insets are molecular structures of 4CzIPN and 5Cz-TRZ.

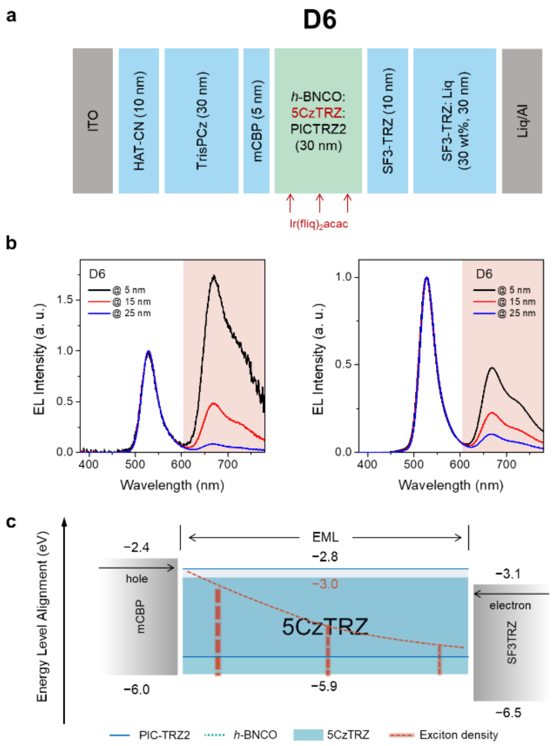

Supplementary Figure 21: a Device structure of D6 with Ir(fliq)2acac films at 5, 15, and 25 nm of the emitting layer (EML). **b** EL spectra of D6 with Ir(fliq)2acac at different positions in EML under low/high current density. **c** Illustration of the exact recombination zone distribution in D6.

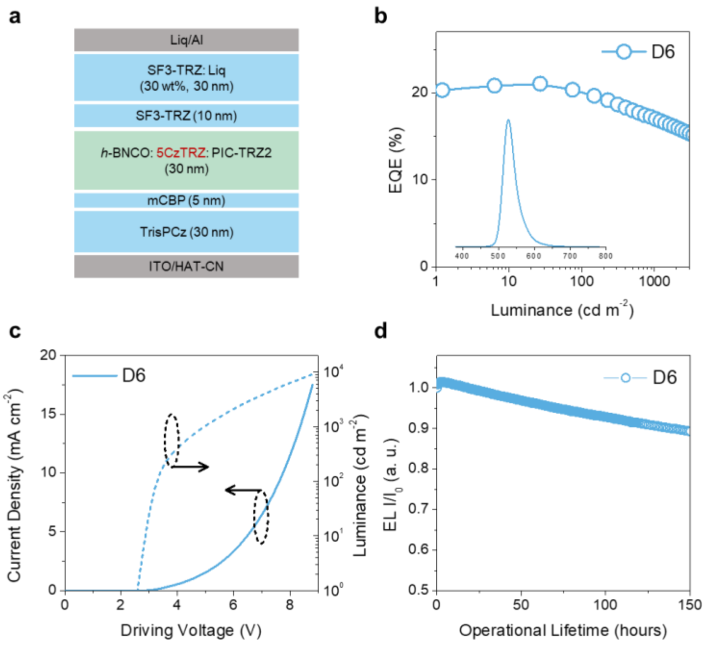

Supplementary Figure 22: OLED performance of 5CzTRZ-based device D6. **a** Device structure. **b** EQE versus luminance curve. The inset is the EL spectrum at 1000 cd m^{-2}. **c** Current density-voltage-luminance curve, and **d** device operational stability.

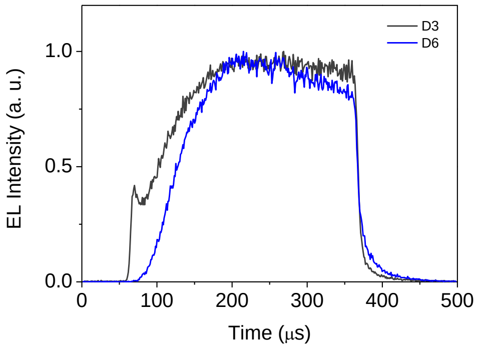

Supplementary Figure 23: Transient EL decay properties of 4CzIPN-based D3 and 5Cz-TRZ-based D6.

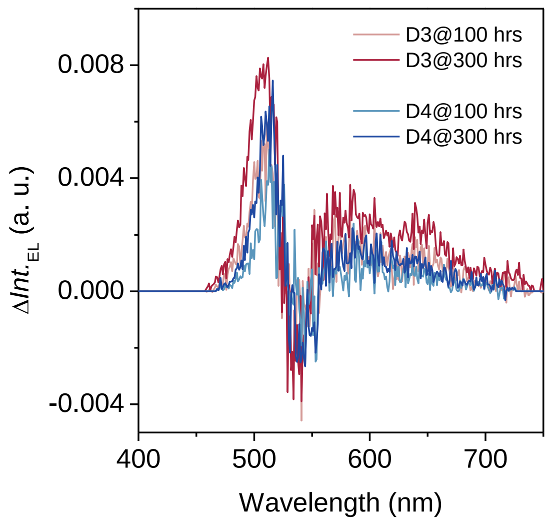

Supplementary Figure 24: The change of EL intensity when D3 and D4 were continuously operating after 100 and 300 hours.

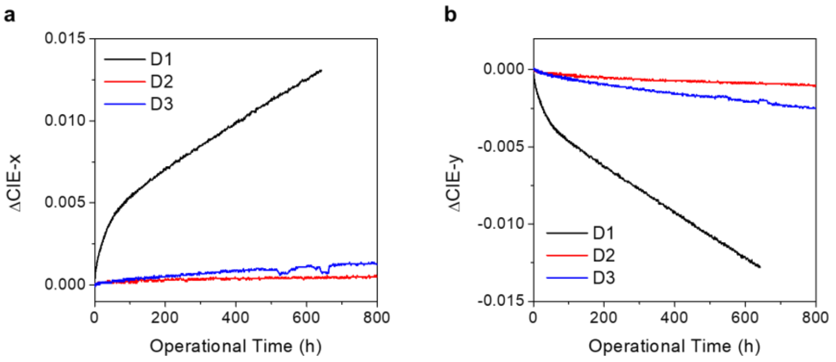

Supplementary Figure 25: The deviation of **a** the CIE-x coordinate, and **b** the CIE-y coordinate in D1-3.

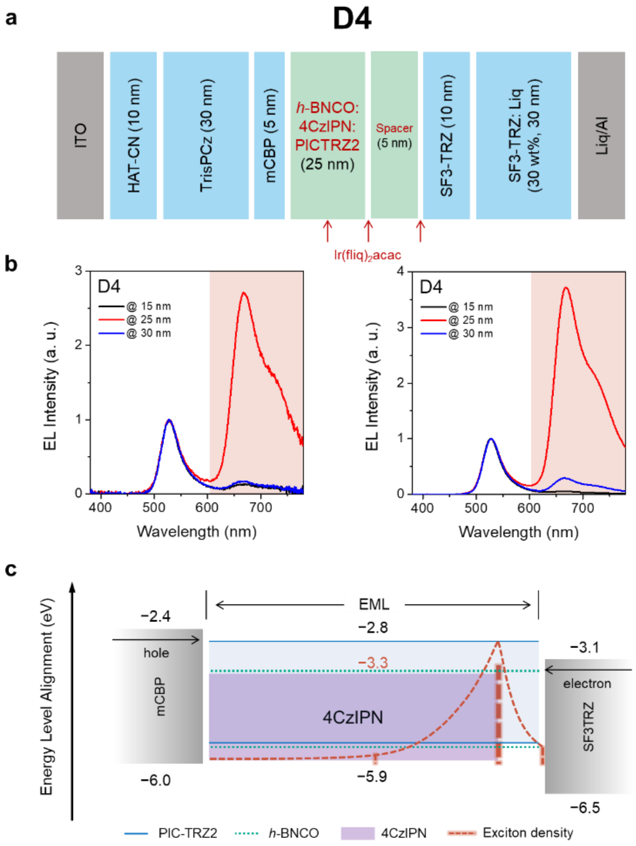

Supplementary Figure 26: a Device structure of D4 with Ir(fliq)$_2$acac films at 5, 15, and 25 nm of the emitting layer (EML). **b** EL spectra of D4 with Ir(fliq)$_2$acac at different positions in EML under low/high current density. **c** Illustration of the exact recombination zone distribution in D4.

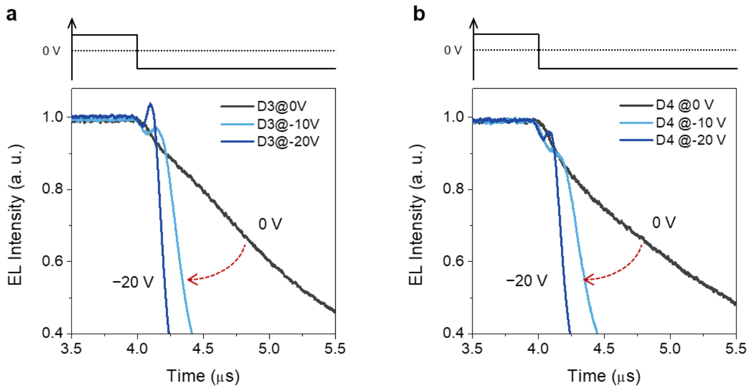

Supplementary Figure 27: Transient EL decay properties of **a** D3, and **b** D4 under pulsed electrical excitation as dependent of different reverse bias voltage (0, −10, and −20 V) after turning off the driving voltage.

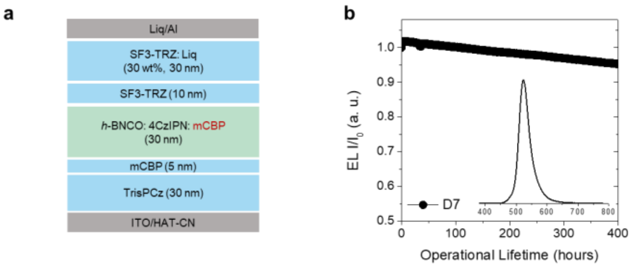

Supplementary Figure 28: OLED performance of mCBP-based hyperfluorescence device D7. **a** Device structure of D7. **b** Device operational stability that measured at the luminance of 1000 cd m^{-2}. The inset is the EL spectrum at 1000 cd m^{-2}.

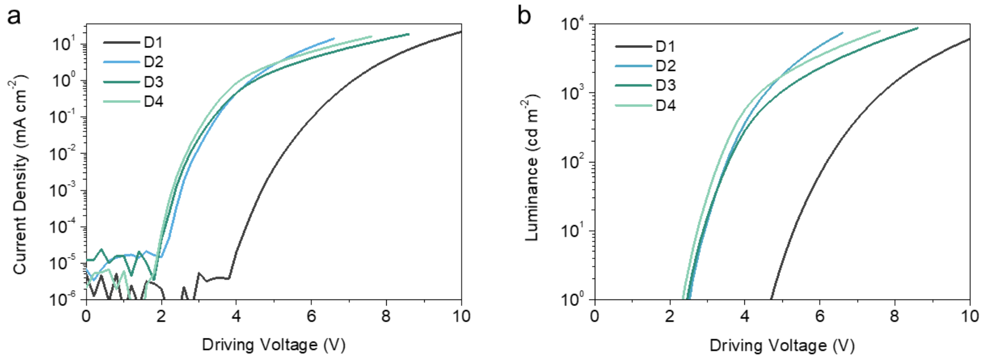

Supplementary Figure 29: a Current density versus driving voltage curve, and **b** luminance versus driving voltage curve of D1-4.

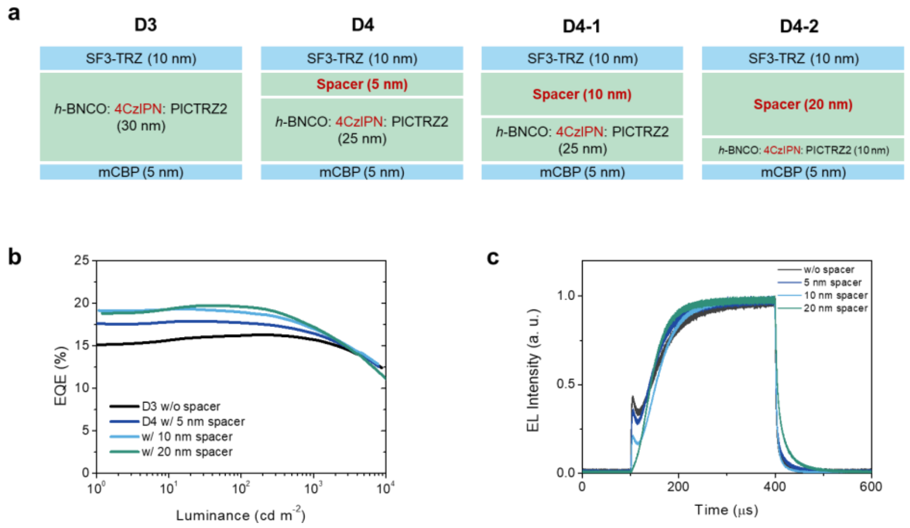

Supplementary Figure 30: a Device structures of OLEDs with different spacer thicknesses. **b** EQE versus luminance curve of these OLEDs. **c** Transient EL decay properties of devices with different spacer thicknesses.

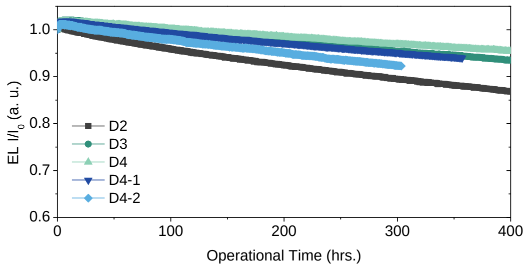

Supplementary Figure 31: Device lifetime of D2, D3, D4, D4-1, and D4-2 at the luminance of 1000 cd m^{-2}.

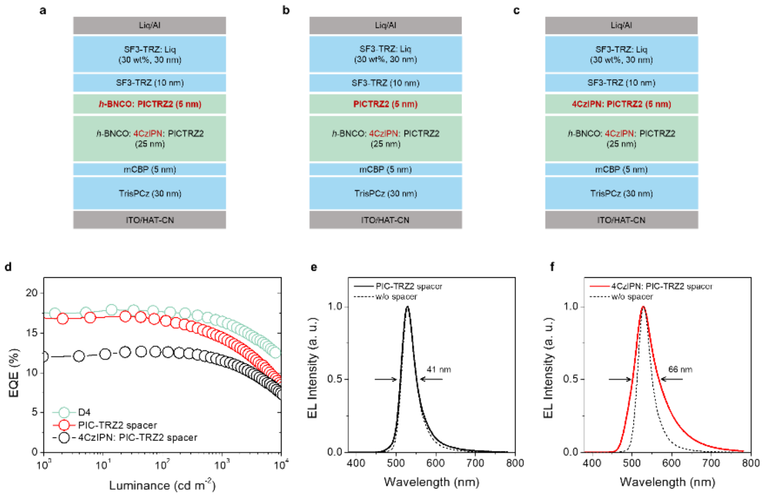

Supplementary Figure 32: Device structures of OLEDs with **a** *h*-BNCO: PIC-TRZ2 spacer (D4), **b** PIC-TRZ2 spacer, and **c** 4CzIPN: PIC-TRZ2 spacer. **d** EQE versus luminance curve. **e** EL spectra of D4 and the device with PIC-TRZ2 spacer. **f** EL spectra of D4 and the device with 4CzIPN: PIC-TRZ2 spacer.

Supplementary Table 1: Photophysical properties of *h*-BNCO-based and PIC-TRZ2-based films.

Film	k_F (10^7 s^{-1})	FRET rate (10^8 s^{-1})	Φ_P	Φ_D	k_{ISC} (10^7 s^{-1})	k_{RISC} (10^5 s^{-1})
1 wt% *h*-BNCO: mCBP	10.3	17.4	0.685	0.106	3.4	2.9
1 wt% *h*-BNCO: PIC-TRZ2	2.9	4.9	0.478	0.293	2.6	5.3
PICTRZ2 neat film	0.11	/	0.110	0.114	0.66	9.0
1 wt% *h*-BNCO: 8 wt% 4CzIPN: PIC-TRZ2	2.5	/	0.403	0.247	3.1	7.4

Supplementary Table 2: Summary of PLQYs of different solid-state films

**Film**	**PLQY (%)**

h-BNCO: mCBP	79.1
h-BNCO: PIC-TRZ2	77.1
h-BNCO: 4CzIPN: PIC-TRZ2	65.0
PIC-TRZ2	22.4
4CzIPN: PIC-TRZ2	34.9
h-BNCO: 5CzTRZ: PIC-TRZ2	72.5

Supplementary Table 3: Summary of device performance with different spacer thicknesses.

Device	Spacer thickness (nm)	EQE_{max} (%)	EQE (%) @1000 cd m^{-2}	Rolloff (%)
D2	0	24.5	18.1	26.1
D3	0	16.3	15.7	3.7
D4	5	17.9	16.6	7.3
D4-1	10	19.4	17.0	12.4
D4-2	20	19.8	17.2	13.1

Supplementary Table 4: Summary of reported device performance with CIE-y around or beyond 0.60.

	Sensitizer of HF	λ_{EL} (nm)	FWHM (nm/eV)	CIE (x, y)	LT95 (hrs)
**This work**	**TADF**	**530**	**38/0.17**	**(0.27, 0.69)**	**437**
Ref. 1 2F-BN	TADF	501	40/-	(0.16, 0.60)	~67[b,c]<br>~20[d]
Ref. 1 3F-BN	TADF	499	38.5/-	(0.20, 0.58)	~7[d]<br>~24[b,c]
Ref. 2 AZA-BN	/	527	30/-	(0.27, 0.69)	~2.5[d]<br>~8.4[b,c]
Ref. 2 AZA-BN	Phosphor	527	30/-	(0.27, 0.69)	~20[d]<br>~67.3[b,c]
Ref. 3 OAB-ABP-1	/	505	33/-	(0.12, 0.63)	/
Ref. 4 BN-DPAC	/	508	49/0.23	(0.16, 0.61)	~1.2[e]<br>~0.4[b,c]
Ref. 5 TCz-B	/	515	30/0.14	(0.16, 0.71)	/
Ref. 5 DACz-B	/	571	44/0.17	(0.47, 0.51)	~1[f]<br>~0.02[b,c]
Ref. 6 DCzBN-Au	/	510	34/0.16	(0.16, 0.67)	27.5[c]
Ref. 7 υ-DABNA-CN-Me	/	504	23/0.112	(0.13, 0.65)	~7[g]<br>~4.7[b,c]
Ref. 8 TRZCzPh-BNCz	/	513	37/-	(0.17, 0.68)	~3.5[h]<br>~1.1[b,c]
Ref. 8 TRZTPh-BNCz	/	513	30/-	(0.17, 0.70)	~3 [h]<br>~0.9[b,c]
Ref. 8 DtCzB-DPTRZ	/	532	40/-	(0.32, 0.63)	~0.1 [h]<br>~0.04[b,c]
Ref. 9 (BzIPr)AuBN	/	513	34/-	(0.18, 0.70)	47.2[c]

Ref. 10 Cz-PTZ-BN	/	520	54/-	(0.26, 0.65)	<0.5[h]<br><0.15[b,c]
Ref. 10 2Cz-PTZ-BN	/	516	56/-	(0.26, 0.63)	<0.5[h]<br><0.15[b,c]
Ref. 11 BNSeSe	/	512	48/-	(0.19, 0.66)	<0.1[c]
Ref. 11 2PXZBN	/	517	49/-	(0.23, 0.67)	~6[c]
Ref. 11 BN3	TADF	558	40/-	(0.41, 0.58)	<0.6[c]
Ref. 12 BN-STO	/	517	34/0.16	(0.19, 0.70)	~1[c]
Ref. 12 BN-XTO	/	516	34/0.16	(0.19, 0.70)	~25[c]
Ref. 13 BN-TP	/	528	36/-	(0.26, 0.70)	<1[i]<br><11.3[b,c]
Ref. 14 *ω*-DABNA	/	512	25/0.114	(0.13, 0.73)	25[c]
Ref. 15 DBNO	/	504	24/-	(0.18, 0.60)	<0.1[h]<br>~0.03[b,c]
Ref. 15 DBNO	TADF	504	27/-	(0.14, 0.53)	<1 [h]<br><0.3[b,c]
Ref. 16 BN-ICz-1	TADF	523	23/0.09	(0.22, 0.74)	~40[d]<br>~135[b,c]
Ref. 16 BN-ICz-2	TADF	523	23/0.09	(0.23, 0.73)	~35[d]<br>~118[b,c]
Ref. 17 tCzphB-Ph	Phosphor	527	24/-	(0.21, 0.75)	~0.25[j]<br>~61.8[b,c]
Ref. 17 tCzphB-Fl	Phosphor	535	26/-	(0.26, 0.72)	~3.5[k]<br>~944[b,c]
Ref. 17 tCzphB-Ph	Phosphor	527	-/-	(0.20, 0.73)	~0.6[l]<br>~128[b,c]
Ref. 17 tCzphB-Fl	Phosphor	535	-/-	(0.26, 0.71)	~22[m]<br>~5725[b,c]
Ref. 18 NBNN2	/	532	38/0.16	(0.29, 0.68)	85.2[c]
Ref. 19 *p*-DBCz	TTA				247.6[n]
Ref. 19 *m*-DBCz	TTA				652.0[n]
Ref. 20 BN-TP-N3	Phosphor	524	34	(0.26, 0.69)	~13[o]

[a] HF indicates hyperfluorescence; [b] LT95/LT90/LT80/LT50 measured at the diverse initial luminance were estimated by using the formula $LT(L_x)=LT(L_0)\times(L_x / L_0)^n$, where L_0 denotes the original luminance, L_x denotes the desired luminance, and n is the lifetime acceleration factor. (For the better comparison, here n was consistent as 1.75); [c] Estimated at luminance of 1000 cd m^{-2}; [d] Estimated at luminance of 2000 cd m^{-2}; [e] Estimated at luminance of 500 cd m^{-2}; [f] Estimated at luminance of 100 cd m^{-2}; [g] Estimated at luminance of 800 cd m^{-2}; [h] Estimated at luminance of 500 cd m^{-2}; [i] Estimated at luminance of 4000 cd m^{-2}; [j] Estimated at luminance of 23300 cd m^{-2}; [k] Estimated at luminance of 24500 cd m^{-2}; [l] Estimated at luminance of 21400 cd m^{-2}; [m] Estimated at luminance of 24000 cd m^{-2}; [n] LT_{98}; [o] Estimated at luminance of 32400 cd m^{-2}.

Supplementary Note

Estimation of photophysical properties. Rate constants, including k_p, k_F , k_{ISC}, and k_{RISC}, were calculated based on the measured quantum yields and transient PL decay lifetime, following the reported methods:[21-23]

$$k_P = \frac{1}{\tau_P} \quad \text{(Eq. S1)}$$

$$k_F = k_P \Phi_P \quad \text{(Eq. S2)}$$

$$k_D = \frac{1}{\tau_D} \quad \text{(Eq. S3)}$$

$k_{ISC} = \frac{k_F}{\Phi_P} - k_F - k_{IC}$ (Eq. S4) ($k_{IC} = \frac{k_F}{\Phi} - k_F$) (Eq. S5)

$k_{RISC} = \frac{k_P k_D}{k_P - k_{ISC}}$ (Eq. S6)

where Φ_P and Φ_D are the prompt and delayed quantum yields, respectively, τ_P and τ_D are the prompt and delayed transient PL lifetime, respectively. k_P, k_F, k_D, k_{ISC}, and k_{RISC} are rate constants of prompt fluorescence, singlet fluorescence radiation, delayed fluorescence, intersystem crossing, and reverse intersystem crossing, respectively. The rate constants were corrected by using the Excel document of the updated method.[23]

For k_{FET}, we fabricated the mCBP neat film, 1 wt% *h*-BNCO: mCBP blend film, PIC-TRZ2 neat film, and 1 wt% *h*-BNCO: PIC-TRZ2 blend film, respectively. For mCBP-based films, we measured the transient PL decay profile at the wavelength of 370 nm, which corresponds to the mCBP region. For PIC-TRZ2-based films, we measured at 440 nm, which is the PIC-TRZ2 region. According to the Eq. R7:[24]

$k_{FET} \approx \frac{1}{\tau_D'} - \frac{1}{\tau_D}$, (Eq. R7)

where k_{FET} is FRET rate, τ_D' and τ_D are the host fluorescence lifetimes with and without the emitter. As obtained from Supplementary Fig. 2, τ_D' and τ_D for mCBP were 0.49 and 3.3 ns, for PIC-TRZ2 were 2.0 and 99 ns, respectively.

Supplementary References

1 Zhang, Y. *et al.* Multi-Resonance Induced Thermally Activated Delayed Fluorophores for Narrowband Green OLEDs. *Angew. Chem. Int. Ed.* **58**, 16912-16917 (2019).

2 Zhang, Y. *et al.* Achieving Pure Green Electroluminescence with CIEy of 0.69 and EQE of 28.2% from an Aza-Fused Multi-Resonance Emitter. *Angew. Chem. Int. Ed.* **59**, 17499-17503 (2020).

3 Ikeda, N. *et al.* Solution-Processable Pure Green Thermally Activated Delayed Fluorescence Emitter Based on the Multiple Resonance Effect. *Adv. Mater.* **32**, e2004072 (2020).

4 Jiang, P. *et al.* Simple Acridan-Based Multi-Resonance Structures Enable Highly Efficient Narrowband Green TADF Electroluminescence. *Adv. Opt. Mater.* **9**, 2100825 (2021).

5 Yang, M. *et al.* Wide-Range Color Tuning of Narrowband Emission in Multi-resonance Organoboron Delayed Fluorescence Materials through Rational Imine/Amine Functionalization. *Angew. Chem. Int. Ed.* **60**, 23142-23147 (2021).

6 Wang, J. *et al.* Metal-Perturbed Multi-Resonance TADF Emitter Enables High-efficiency and Ultralow Efficiency Roll-off Non-Sensitized OLEDs with Pure Green Gamut. *Adv. Mater.* **35**, 2208378 (2023).

7 Oda, S. *et al.* Development of Pure Green Thermally Activated Delayed Fluorescence Material by Cyano Substitution. *Adv. Mater.* **34**, e2201778 (2022).

8 Liu, Y. *et al.* Space-Confined Donor-Acceptor Strategy Enables Fast Spin-Flip of Multiple Resonance Emitters for Suppressing Efficiency Roll-Off. *Angew. Chem. Int. Ed.* **61**, e202210210 (2022).

9 Cai, S. *et al.* Gold(I) Multi-Resonance Thermally Activated Delayed Fluorescent Emitters for Highly Efficient Ultrapure-Green Organic Light-Emitting Diodes. *Angew. Chem. Int. Ed.*, **61**, e202213392 (2022).

10 Liu, F. *et al.* Highly Efficient Asymmetric Multiple Resonance Thermally Activated Delayed Fluorescence Emitter with EQE of 32.8 % and Extremely Low Efficiency Roll-Off. *Angew. Chem. Int. Ed.* **61**, e202116927 (2022).

11 Hu, Y. X. *et al.* Efficient selenium-integrated TADF OLEDs with reduced roll-off. *Nat. Photonics.* **16**, 803-810 (2022).

12 Hu, Y. X. *et al.* Peripherally Heavy-Atom-Decorated Strategy Towards High-Performance Pure Green Electroluminescence with External Quantum Efficiency over 40% *Angew. Chem. Int. Ed.* **62**, e202302478 (2023).

13 Xu, Y. *et al.* Constructing Organic Electroluminescent Material with Very High Color Purity and Efficiency Based on Polycyclization of the Multiple Resonance Parent Core. *Angew. Chem. Int. Ed.* **61**, e202204652 (2022).

14 Uemura, S. *et al.* Sequential Multiple Borylation Toward an Ultrapure Green Thermally Activated Delayed Fluorescence Material. *J. Am. Chem. Soc.* **145**, 1505-1511 (2022).

15 Cai, X. *et al.* Achieving 37.1% Green Electroluminescent Efficiency and 0.09 eV Full Width at Half Maximum Based on a 3 Boron-Oxygen-Nitrogen Embedded Polycyclic Aromatic System. *Angew. Chem. Int. Ed.* **61**, e202200337 (2022).

16 Zhang, Y. *et al.* Fusion of Multi-Resonance Fragment with Conventional Polycyclic Aromatic Hydrocarbon for Nearly BT.2020 Green Emission. *Angew. Chem. Int. Ed.* **61**, e202202380 (2022).
17 Liu, J. *et al.* Toward a BT.2020 green emitter through a combined multiple resonance effect and multi-lock strategy. *Nat. Commun.* **13**, 4876 (2022).
18 Luo, S. *et al.* Regulation of Multiple Resonance Delayed Fluorescence via Through-Space Charge Transfer Excited State towards High-Efficiency and Stable Narrowband Electroluminescence. *Angew. Chem. Int. Ed.* **62**, e2023109 (2023)
19 Cai, X. *et al.* Multi-Resonance Building-Block-Based Electroluminescent Material: Lengthening Emission Maximum and Shortening Delayed Fluorescence Lifetime. *Angew. Chem. Int. Ed.* **62**, e2023041 (2023)
20 Wang, Q. *et al.* Precise Regulation of Emission Maxima and Construction of Highly Efficient Electroluminescent Materials with High Color Purity. *Angew. Chem. Int. Ed.* **62**, 202301930 (2023)
21 Masui, K.; Nakanotani, H.; Adachi, Analysis of exciton annihilation in high-efficiency sky-blue organic light-emitting diodes with thermally activated delayed fluorescence. *Org. Electron.* **14**, 2721 (2013)
22 Sano, Y., *et al.* One-Shot Construction of BN-Embedded Heptadecacene Framework Exhibiting Ultra-narrowband Green Thermally Activated Delayed Fluorescence. *J. Am. Chem. Soc.* **145**, 11504 (2023)
23 Tsuchiya, Y., *et al.* Exact Solution of Kinetic Analysis for Thermally Activated Delayed Fluorescence Materials. *J. Phys. Chem. A* **125**, 8074 (2021)
24 Ghenuche, P., *et al.* Nanophotonic Enhancement of the Förster Resonance Energy-Transfer Rate with Single Nanoapertures. *Nano Lett.* **14**, 4707 (2014)
